# The emerging role of digital dentistry in managing molar-incisor hypomineralization: A perspective

**DOI:** 10.1371/journal.pdig.0001406

**Published:** 2026-05-11

**Authors:** Cinthya Jeanette Arias Guillén, Ulysses de Toledo Monteiro, Anna Laura Doalto, João Sarmento Pereira Neto, Regina Maria Puppin-Rontani, Aline Castilho

**Affiliations:** 1 Departamento‌‌ de Saúde Coletiva, Odontopediatria e Ortodontia, Faculdade de Odontologia de Piracicaba, Universidade Estadual de Campinas, Piracicaba, São Paulo, Brasil; 2 Departamento‌‌ de Odontologia Restauradora, Faculdade de Odontologia de Piracicaba, Universidade Estadual de Campinas, Piracicaba, São Paulo, Brasil; 3 Faculdade de Odontologia de Piracicaba, Universidade Estadual de Campinas, Piracicaba, São Paulo, Brasil; 4 Departamento de Saúde Coletiva, Odontopediatria e Ortodontia, Faculdade de Odontologia de Piracicaba, Universidade Estadual de Campinas, Piracicaba, São Paulo, Brasil; 5 Department of Pediatric Dentistry, Indiana University School of Dentistry, Indianapolis, Indiana, United States of America; Henry Ford Health System, UNITED STATES OF AMERICA

## Abstract

Molar-incisor hypomineralization (MIH) remains one of the most challenging conditions in pediatric dentistry, with fragile enamel that compromises structural integrity, patient comfort, and the longevity of traditional restorative treatments. The unpredictable bonding to hypomineralized substrates results in frequent restoration failures, reinforcing the need for new approaches. In this opinion piece, we argue that digital dentistry offers promising strategies to improve precision, durability, and patient-centered care in MIH management. Drawing on a clinical case used as a proof of concept to illustrate feasibility, we describe a fully digital workflow including intraoral scanning, CAD/CAM design, and 3D-printed indirect restoration. Digital workflows may also enrich dental education by familiarizing students with emerging technologies. At the same time, we acknowledge key barriers such as cost, accessibility, and the clinician learning associated with digital adoption. Digital solutions hold promise as part of a broader strategy to improve care for patients with MIH.

## Introduction

Molar-incisor‌‌ hypomineralization (MIH) is a developmental enamel defect affecting first permanent molars and often permanent incisors; with occasional involvement of the canines. Clinically, it manifests as demarcated opacities, post-eruptive enamel breakdown, hypersensitivity, and a higher caries risk [1]. Although some studies suggest an apparent increase in prevalence, this trend may reflect improved recognition and diagnostic awareness rather than a true epidemiologic rise [2]. Its etiology is multifactorial, with prenatal, perinatal, and postnatal factors implicated; recent evidence also points to a genetic component [3].

MIH-affected enamel is more porous and contains higher protein levels, which compromise adhesion and contribute to restoration failure [4]. These challenges highlight the need for approaches that go beyond material substitution [5]. Thus, digital workflows may represent such an opportunity by enabling more precise, efficient, and patient-centered restorative care.

## A clinical case as proof of concept

**Fig 1:**
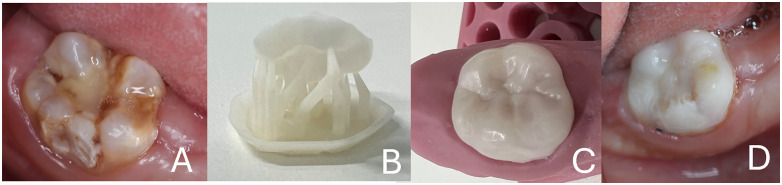
Composite illustration of initial condition (A), digital workflow (B,C) and final restoration (D).

A 10-year-old male presented with severe MIH, hypersensitivity and structural loss of #30. Previous conventional restorations had failed. Informed consent was obtained to use anonymized images for educational purpose. A fully digital workflow was used: selective preparation preserved, intraoral scanning (Primescan, Dentsply Sirona, Germany), CAD/CAM design (Exocad, Germany) and a 3D-printed indirect resin restoration (Photon Mono 2, Anycubic, China). Cementation followed a standard adhesive protocol. The patient reported immediate improvement in comfort and stable function at the one-year follow-up. We explicitly state that this single case is illustrative and not generalizable (Fig 1).

## Why digital dentistry may change the game

The challenges associated with MIH (hypersensitivity, compromised enamel, difficulty achieving adequate anesthesia, frequent restoration failure) [6] make digital workflows an appealing complementary approach [7]. Digital impressions can improve accuracy, even on irregular enamel surfaces, and reduce chair time for pediatric patients [8,9]. Indirect restorations produced via CAD/CAM or 3D printing may provide improved marginal adaptation compared with some direct techniques [10,11]. Digital workflows also support dental education by familiarizing students with equipment and processes increasingly present in clinical practice [12]. However, digital dentistry is not a universal solution. Scanners may struggle with saliva, limited mouth opening, or deep occlusal anatomy [13,14], and long-term behavior of 3D-printed materials is still under evaluation [15]. Implementation barriers such as cost, training needs, and access to equipment must also be considered. Thus, digital approaches should be viewed as promising and context-dependent rather than definitive alternatives to conventional care.

## Outcome assessment and limitations

Post-treatment evaluation considered hypersensitivity reduction, restoration integrity, and functional performance. However, we emphasize that evidence from a single case must be interpreted cautiously.

## Final considerations

MIH poses substantial clinical and educational challenges. Digital dentistry offers promising strategies to enhance precision, comfort, and workflow efficiency in managing affected teeth. Yet meaningful adoption will require addressing financial, infrastructural, and training barriers. Continued research and educational integration will be essential for determining how digital workflows can best support care for children with MIH.
